# Mutations in Efflux Pump Rv1258c (Tap) Cause Resistance to Pyrazinamide, Isoniazid, and Streptomycin in *M. tuberculosis*

**DOI:** 10.3389/fmicb.2019.00216

**Published:** 2019-02-19

**Authors:** Jiayun Liu, Wanliang Shi, Shuo Zhang, Xiaoke Hao, Dmitry A. Maslov, Kirill V. Shur, Olga B. Bekker, Valery N. Danilenko, Ying Zhang

**Affiliations:** ^1^Department of Clinical Laboratory, Xijing Hospital, Fourth Military Medical University, Xi’an, China; ^2^Department of Molecular Microbiology and Immunology, Bloomberg School of Public Health, Johns Hopkins University, Baltimore, MD, United States; ^3^Laboratory of Bacterial Genetics, Vavilov Institute of General Genetics, Russian Academy of Sciences, Moscow, Russia

**Keywords:** drug resistance, efflux pump, mutations, tuberculosis, pyrazinamide

## Abstract

Although drug resistance in *Mycobacterium tuberculosis* is mainly caused by mutations in drug activating enzymes or drug targets, there is increasing interest in the possible role of efflux in causing drug resistance. Previously, efflux genes have been shown to be upregulated upon drug exposure or implicated in drug resistance in overexpression studies, but the role of mutations in efflux pumps identified in clinical isolates in causing drug resistance is unknown. Here we investigated the role of mutations in efflux pump Rv1258c (Tap) from clinical isolates in causing drug resistance in *M. tuberculosis.* We constructed point mutations V219A and S292L in Rv1258c in the chromosome of *M. tuberculosis* and the point mutations were confirmed by DNA sequencing. The susceptibility of the constructed *M. tuberculosis* Rv1258c mutants to different tuberculosis drugs was assessed using conventional drug susceptibility testing in 7H11 agar in the presence and absence of efflux pump inhibitor piperine. A C14-labeled PZA uptake experiment was performed to demonstrate higher efflux activity in the *M. tuberculosis* Rv1258c mutants. Interestingly, the V219A and S292L point mutations caused clinically relevant drug resistance to pyrazinamide (PZA), isoniazid (INH), and streptomycin (SM), but not to other drugs in *M. tuberculosis.* While V219A point mutation conferred low-level drug resistance, the S292L mutation caused a higher level of resistance. Efflux inhibitor piperine inhibited INH and PZA resistance in the S292L mutant but not in the V219A mutant. The S292L mutant had higher efflux activity for pyrazinoic acid (the active form of PZA) than the parent strain. We conclude that point mutations in the efflux pump Rv1258c in clinical isolates can confer clinically relevant drug resistance, including PZA resistance, and could explain some previously unaccounted drug resistance in clinical strains. Future studies need to take efflux mutations into consideration for improved detection of drug resistance in *M. tuberculosis* and address their role in affecting treatment outcome *in vivo*.

## Introduction

Multidrug resistant (MDR) tuberculosis (MDR-TB), defined by resistance to at least the two frontline drugs isoniazid (INH) and rifampin (RIF), poses a significant challenge to effective treatment and control of the disease. In 2015, there were at least 480,000 cases of MDR-TB and about 9.5% of MDR-TB cases were extensively drug resistant TB, or XDR-TB (WHO, 2018). Drug resistance in the causative agent *Mycobacterium tuberculosis* is mainly caused by mutations affecting enzymes involved in drug activation or by alterations or overexpression of drug targets (Zhang and Yew, 2009). However, efflux pumps, which have been found to cause antibiotic resistance in many other bacteria (Li and Nikaido, 2009) and also non-tuberculous mycobacteria (Liu et al., 1996; Ainsa et al., 1998) have only recently been shown to be involved in clinical drug resistance in *M. tuberculosis* in the case of clofazimine and bedaquiline (Andries et al., 2014; Hartkoorn et al., 2014).

The efflux protein Rv1258c, also called Tap or P55, was previously shown to be involved in resistance to different drugs in overexpression studies involving *Mycobacterium megmatis* and *Mycobacterium bovis* BCG (Ramon-Garcia et al., 2012; Shur et al., 2017). For example, overexpression of Rv1258c was shown to confer resistance to INH, RIF, ethambutol, PAS, and ethambutol in *M. bovis* BCG (Ramon-Garcia et al., 2012). Additionally, Jiang et al. (2008) showed that Rv1258c was overexpressed upon exposure to INH and RIF in MDR-TB clinical isolates and hypothesized that its overexpression may cause drug resistance in clinical TB strains. Furthermore, various single nucleotide polymorphisms (SNPs) in different efflux pump genes Rv0194, Rv1217, Rv1258c, Rv1273, Rv1877, Rv1250, and Rv2688 in XDR-TB clinical isolates have recently been identified (Kanji et al., 2017). However, the relevance of these SNPs in causing drug resistance in clinical strains has been unclear. In this study, we identified SNPs in the efflux gene *rv1258c* (*tap*) from clinical isolates of *M. tuberculosis* and then evaluated the significance of the SNPs in causing clinically relevant drug resistance by introducing these point mutations into the genome of the isogenic strain of *M. tuberculosis*. We demonstrate that mutations (V219A, S292L) in efflux pump Tap Rv1258c identified in clinical strains can cause clinically relevant drug resistance in *M. tuberculosis.*

## Materials and Methods

### Bacterial Strains, Plasmids and Drugs

*Mycobacterium tuberculosis* H37Ra was grown at 37°C in Middlebrook 7H9 liquid medium or on 7H11 agar plates supplemented with 10% (v/v) albumin–dextrose–catalase (ADC, Becton Dickinson, Sparks, MD, United States) plus 0.5% (v/v) glycerol, 0.25% (v/v) Tween 80. Plasmids p2NIL and pGOAL19 used in this study were obtained from Addgene (Cambridge, MA, United States). isoniazid (INH), rifampicin (RIF), streptomycin (SM), ethambutol, pyrazinamide (PZA), levofloxacin, amikacin, cycloserine, p-aminosalicylic acid (PAS), clofazimine (CFZ), tetracycline, linezolid, clarithromycin, and piperine were purchased from Sigma-Aldrich (St Louis, MO, United States). The drugs were dissolved in DMSO and further diluted to obtain the desired concentrations in culture media for drug susceptibility testing (see below).

### Construction of Rv1258c Point Mutation Mutants by Homologs Recombination

The Rv1258c mutants were constructed in *M. tuberculosis* H37Ra by homologous recombination as described previously (Parish and Stoker, 2000). The mutant construction and drug susceptibility testing were performed in BSL2 laboratory following institutional biosafety procedures. The reason we used the avirulent *M. tuberculosis* H37Ra strain is because it is a good surrogate of its virulent parent strain H37Rv in terms of drug susceptibility profiles (Heinrichs et al., 2018). Briefly, the Rv1258c gene was amplified with its adjacent 1 kb fragment on both sides from *M. tuberculosis* genomic DNA and cloned into p2NIL plasmid vector. Then, the mutated constructs Rv1258c V219A and S292L were obtained by QuikChange II XL site-directed mutagenesis kit (Agilent, Santa Clara, CA, United States) with primers TPV219AF: 5′-TTCGCCTGGAACCTGCGGGTATTGCGCACCC-3′, TPV219AR: 5′-CCAGGCGAAGCGCAGCCCCTCGGCGATCCC-3′, TPS292LR: 5′-CCCCGTCGCGTGACCATGCTGACCGCGGTTCTTACCCTGGG-3′, and TPS292LR: 5′-CCCAGGGTAAGAACCGCGGTCAGCATGGTCACGCGACGGGG-3′. pGOAL19 cassette was cloned into the PacI site of p2NIL containing the mutant (V219A and S292L) version of the Rv1258c gene to form suicide delivery constructs. The suicide delivery plasmid DNA was subjected to 100 mJ/cm2 of UV irradiation followed by addition of 200 μl of electrocompetent mycobacteria. Electroporation was performed with the parameters 2.5 kV, 1000 Ω, 25 mF. The electroporated cells were added with 200 μl 7H9-Tween 80 recovery medium and incubated at 37°C for 24 h followed by plating onto 7H11/ADC plates containing kanamycin at 20 μg/ml, hygromycin at 50 μg/ml, and X-Gal at 50 μg/ml. The plates were incubated at 37°C for 4–5 weeks until colonies appeared. Single cross-over transformants were picked and re-streaked onto fresh 7H11/ADC without any antibiotics and incubated at 37°C for 2–3 weeks. A loopful of bacteria from the plates was resuspended in 1 ml of 1-mm glass beads and 3 mL of 7H9/ADC/Tween 80 and vortexed vigorously. Serial dilutions were plated onto plates containing X-gal with and without sucrose at 2% (w/v) and incubated for 4 weeks until the potential double cross-over transformants appeared. The kanamycin-sensitive colonies were screened using colony-PCR followed by DNA sequencing to confirm the desired point mutation being constructed in the genome of *M. tuberculosis* H37Ra.

### Drug Susceptibility Testing and Isoniazid/Piperine Combination Studies

The Rv1258c V219A and S292L mutants and parent strain *M. tuberculosis* H37Ra were grown in 7H9/ADC/Tween 80 medium at 37°C for 2–3 weeks [approximately 1 × 10^8^colony-forming units (CFU)/ml] when they were diluted in serial 10-fold dilutions and inoculated onto 7H11 agar plates containing different concentrations of drugs in triplicate. The following drug concentrations were used: SM (0.5, 1, 2, 4, and 8 μg/ml). INH (0.125, 0.25, 0.5, 1, 2, 4, 8, 16, 32, and 64 μg/ml), PZA (50, 100, 200, 400, 800, and 1600 μg/ml, pH 6.0), PAS (0.25, 0.5, 1, 2, 4, 8, 16, 32, 64, and 128 μg/ml), RIF (0.25, 0.5, 1, 2, and 4 μg/ml), ethambutol (1, 2, 4, 8, and 16 μg/ml), levofloxacin (0.25, 0.5, 1, 2, and 4 μg/ml), amikacin (0.5, 1, 2, 4, and 8 μg/ml), cycloserine (5, 10, 20, 40, and 80 μg/ml), CFZ (0.125, 0.25, 0.5, 1, and 2 μg/ml), tetracycline (0.5, 1, 2, 4, 8, 16, 32, 64, and 128 μg/ml), linezolid (0.25, 0.5, 1, 2, and 4 μg/ml), and clarithromycin (0.125, 0.25, 0.5, and 1, 2 μg/ml). The plates were incubated at 37°C in 5% CO_2_ for 3–4 weeks. The INH/piperine combination study was performed on the Rv1258c V219A and S292L mutants with different concentrations of INH tested in the presence of increasing concentrations of piperine (0, 5, 10, 20, and 40 μg/ml).

### PZA Uptake and Accumulation Experiments

Two-week-old cultures of Rv1258c V219A and S292L mutants of *M. tuberculosis* H37Ra, grown in in7H9/ADC/Tween 80 medium, were harvested and the cell pellets were resuspended in 7H9 medium (pH 6.6) at 5 × 10^8^ cells/ml. The PZA uptake and accumulation study was performed as described previously (Zhang et al., 1999). Briefly, [^14^C]PZA, purchased from Vitrax (Placentia, CA, United States), was added to the cell suspensions to a concentration of 1 μCi/ml and the cell mixtures were incubated at 37°C. At different time points, 50 μl portions were removed and washed with 1 × PBS buffer (pH 6.6) with 0.1M LiCl by filtration on 0.45 μm-pore-size nitrocellulose filters by using a vacuum pump. The amount of radioactivity associated with the bacterial cells was determined by autoradiography and scintillation counting.

## Results and Discussion

### Identification of Point Mutations in Rv1258c From Clinical Isolates of *M. tuberculosis* and Construction of Rv1258c Point Mutations in *M. tuberculosis*

To determine possible roles of efflux pumps in causing drug resistance, we performed a database search for mutations in the efflux pump Rv1258c (Tap) among clinical isolates whose whole genome sequences are deposited in the GenBank NCBI database. Several single nucleotide polymorphisms were found in the efflux gene Rv1258c (D23V, V219A, and S292L) in different clinical isolates of *M. tuberculosis*, which may affect the function of the efflux protein. This is consistent with the identification of Rv1258c P369T and G391R in XDR-TB clinical isolates from sequenced genomes of *M. tuberculosis* strains from Pakistan (Kanji et al., 2017).

To address the role of the identified point mutations in Rv1258c, we attempted to construct the point mutations D23V, V219A, and S292L by site-directed mutagenesis by PCR and the altered mutant sequences were confirmed to be correct by DNA sequencing. We were able to successfully construct Rv1258c point mutations V219A and S292L, but not D23V (for unknown reasons) in *M. tuberculosis* H37Ra. It is worth noting that the constructed Rv1258c point mutation V219A and S292L mutant strains had no growth defect or altered morphology as seen in Rv1258c deletion mutant strain (Ramon-Garcia et al., 2012). Therefore, we evaluated the susceptibility of Rv1258c point mutation V219A and S292L mutants to various drugs as described below.

### Rv1258c Point Mutations Confer Resistance to PZA, INH, and SM

**FIGURE 1:**
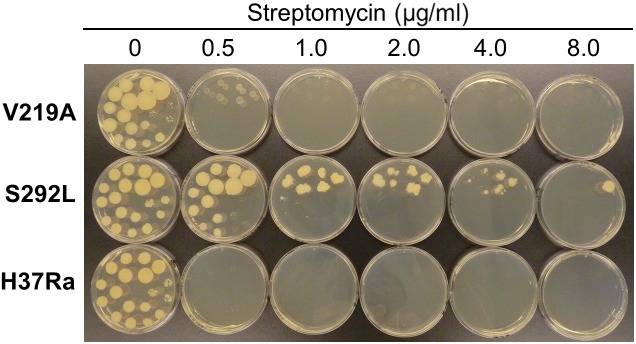
The Rv1258c S292L mutant of *Mycobacterium tuberculosis* has higher level of resistance to streptomycin (SM) than the V219 mutant.

**FIGURE 2:**
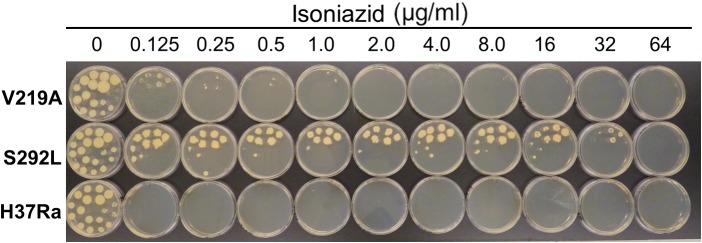
The Rv1258c S292L mutant of *M. tuberculosis* is highly resistant to isoniazid (INH), while the V219A mutant is only slightly resistant.

**FIGURE 3:**
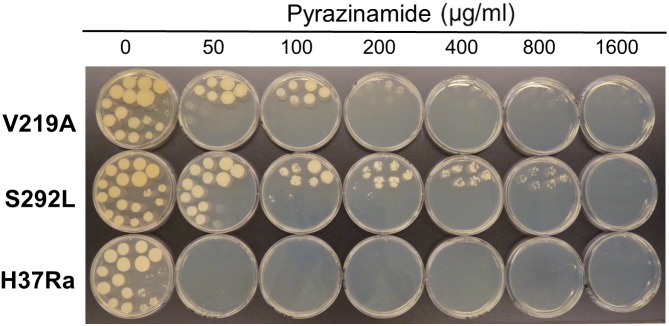
The Rv1258c S292L mutant of *M. tuberculosis* has a higher level of PZA resistance than the V219A mutant.

**FIGURE 4:**
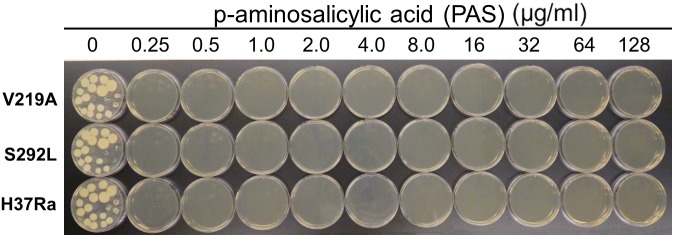
The Rv1258c S292L and V219A mutants of *M. tuberculosis* remain susceptible to PAS.

The results of drug susceptibility testing showed that Rv1258c S292L mutant was more resistant to SM, INH, and PZA than the parent strain H37Ra (Table 1). However, Rv1258c V219A point mutation caused a lower level of resistance to the above drugs than the S292L mutation. It is noteworthy that while V219A mutation only caused a marginal level of INH resistance (MIC = 0.125 μg/ml), S292L mutation caused a very high level of INH resistance (MIC = 32 μg/ml) (Table 1). This latter finding is significant as it offers a possible alternative mechanism of INH resistance in addition to mutations in *katG* (Zhang et al., 1992; Zhang and Yew, 2009) or *inhA* (Banerjee et al., 1994). Introduction of the V219A and S292L mutations in *M. tuberculosis* did not alter the susceptibility to other drugs including RIF, ethambutol, levofloxacin, amikacin, cycloserine, PAS, CFZ, tetracycline, linezolid and clarithromycin (Figure 1–4 and Table 1). Our finding that Rv1258c S292L point mutation conferred resistance to SM and INH is consistent with the previous observation of overexpression of Rv1258c (Ramon-Garcia et al., 2012). However, the Rv1258c S292L and V219A mutations did not alter susceptibility to other drugs such as tetracycline, RIF, EMB, CFZ, while previous studies have demonstrated that overexpression of Rv1258c could cause resistance to these drugs (Ainsa et al., 1998; Ramon-Garcia et al., 2009, 2012). One possibility for such a discrepancy is that the point mutations at S292L and V219A cause differential binding to different drugs such that they have differential effects on causing selective drug resistance to some but not other drugs, as seen in overexpression study of the wild-type functional protein Rv1258c (Ramon-Garcia et al., 2009, 2012).

**Table 1:** Susceptibility of *M. tuberculosis* efflux pump Rv1258c point mutation mutants to different TB drugs.

Drugs	MIC breakpoints	Drug susceptibility results#	
	H37Ra (μg/ml)	Rv1258c V219A	Rv1258c S292L
Isoniazid	0.05	R (MIC 0.125 μg/ml)	R (MIC 32 μg/ml)
Pyrazinamide	50	R (MIC 100 μg/ml)	R (MIC 800 μg/ml)
Streptomycin	0.5	S	R (MIC 4.0 μg/ml)
Rifampin	1.0	S	S
Ethambutol	5.0	S	S
Amikacin	1.0	S	S
Cycloserine	10	S	S
p-aminosalicylic acid (PAS)	0.20	S	S
Clofazimine	0.20	S	S
Tetracycline	8.0	S	S
Linezolid	0.5	S	S
Clarithromycin	2.0	S	S


*# S indicates “susceptible”; R indicates “resistant”*.

Previous studies that evaluated drug resistance conferred by overexpression of Rv1258c did not test PZA, presumably because of the well-known problem with its susceptibility testing (Zhang and Mitchison, 2003). Here, it is of interest to note that introducing the S292L point mutation in Rv1258c into the chromosome of *M. tuberculosis* conferred a high level of PZA resistance (800 μg/ml), whereas the V219A mutation caused a low level of PZA resistance (100–200 μg/ml) (Figure 3). Although PZA resistance is mostly caused by *pncA* mutations (Zhang Y. et al., 2013) and less commonly by *rpsA* (Shi et al., 2011)*, panD* (Zhang S. et al., 2013), and *clpC1* (Yee et al., 2017; Zhang S. et al., 2017) mutations, some PZA-resistant *M. tuberculosis* strains without mutations in the above known genes do exist (Cheng et al., 2000) (Zhang Y, unpublished). Recently, we have shown that overexpression of efflux proteins Rv0191, Rv3756c, Rv3008, and Rv1667c could all confer low-level PZA resistance in *M. tuberculosis* (Zhang Y. et al., 2017), indicating a role of efflux in PZA resistance. However, not all efflux pumps are involved in PZA resistance as overexpression of efflux pump DrrAB in *M. tuberculosis* did not confer PZA resistance (Zhang Y, unpublished). Here it is worth noting that by constructing point mutations in the genome of the efflux pump gene *rv1258c*, we were able to convincingly demonstrate that the S292L mutation is indeed causative of PZA resistance. This finding suggests that mutations in Rv1258c could be a potential new mechanism of PZA resistance in clinical isolates without known structural gene (*pncA, rpsA, panD*) mutations. Future studies are needed to determine how frequently such mechanisms of PZA resistance mediated by mutations in Rv1258c occur in clinical isolates. Although we were able to demonstrate that the point mutations in Rv1258c confer drug resistance in *M. tuberculosis*, it remains to be determined if the mutations caused the elevated drug resistance phenotype through affecting the stability (Zhu et al., 2011), expression levels (Kardan-Yamchi et al., 2019), or drug affinity (Cloete et al., 2018) of the mutant Rv1258c protein. Future studies are needed to address these possibilities.

### Piperine Inhibits the INH and PZA Resistance in the Rv1258c S292L Mutant

**FIGURE 5:**
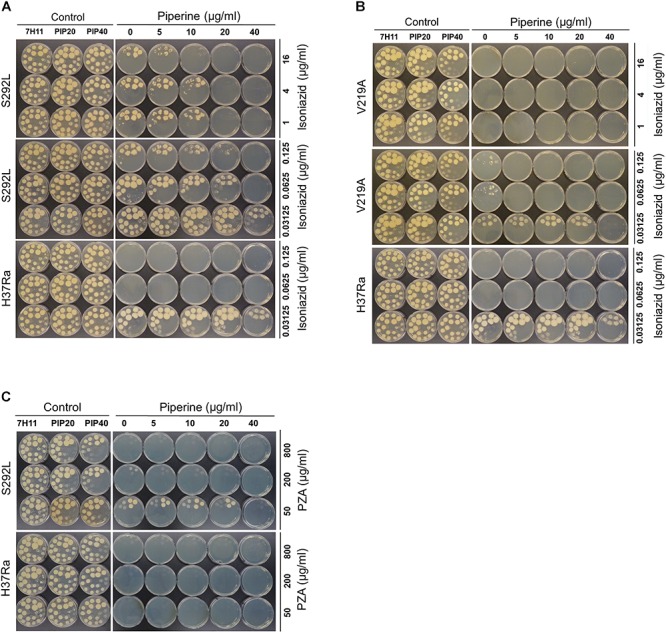
Effect of efflux pump inhibitor Piperine on INH resistance and PZA resistance in Rv1258c mutants of *M. tuberculosis.* Piperine at 40 mg/ml but at lower concentrations inhibited or reduced the level of INH resistance **(A)** and PZA resistance **(C)** in the Rv1258c S292L mutant, but not in the V219A mutant **(B)**.

Piperine is a known inhibitor of efflux pump protein Rv1258c in *M. tuberculosis* (Sharma et al., 2010). To determine if the S292L point mutation mediated higher level of resistance is due to elevated efflux activity of the Rv1258c S292L mutant protein, we tested if piperine could antagonize the INH and PZA resistance mediated by the Rv1258c S292L mutant protein. The piperine/INH or piperine/PZA combination study showed that piperine reduced both INH and PZA resistance or increased INH and PZA susceptibility in the Rv1258c S292L mutant *M. tuberculosis* strain (Figure 5A,C), but much less so in the V219A mutant strain, presumably due to the much weaker efflux activity of the V219A mutant (Figure 5B) compared with the S292L mutant. The inhibitory effect of piperine on reducing INH resistance in the V219A mutant could only be observed at a higher piperine concentration (40 μg/ml) at a low INH concentration of 0.0325 μg/ml (Figure 5B). The results indicate that the higher level of INH or PZA resistance in the Rv1258c S292L mutant is caused by higher efflux activity of the S292L mutant protein that could be inhibited by piperine.

### PZA-Resistant Rv1258c S292L Mutant Accumulates Less Drug POA

**FIGURE 6:**
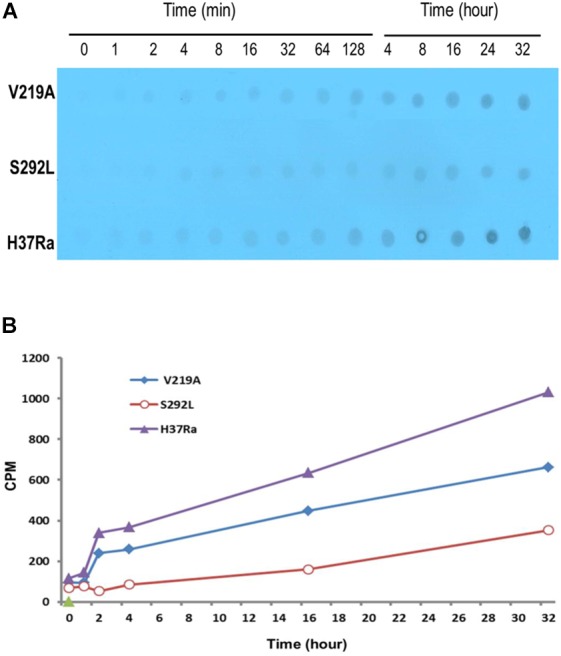
Comparison of PZA/POA accumulation in Rv1258c mutants of *M. tuberculosis*. [^14^C]PZA was added to 5 × 10^9^ *M. tuberculosis* H37Ra and the Rv1258c mutant strains at 1 mCi/ml at pH 6.6. At different times after PZA addition, portions of bacterial cells were removed and washed by filtration using PBS (pH 6.6) as described in Methods. The results of PZA/POA accumulation are shown by autoradiography **(A)** and scintillation counting **(B)**. The S292L mutant accumulated less PZA/POA than the V219A mutant, while both mutants accumulated less drug than the parent strain *M. tuberculosis* H37Ra as shown in both **(A,B)**.

It has been shown that [^14^C]PZA is converted to [^14^C]POA in *M. tuberculosis* and accumulates in the cell to some extent due to a weak efflux of POA (Zhang et al., 1999). To determine if Rv1258c V219A and S292L mutants, which are confirmed to be PZA-resistant in susceptibility testing (Figure 3), can pump [^14^C]PZA out of the cell more effectively, we performed the [^14^C]PZA uptake experiment comparing the amount of [^14^C]POA accumulated in the cell in the Rv1258c mutants and that of the control parent strain. It was found that the intracellular concentration of POA in Rv1258c mutants was lower than that of PZA-susceptible *M. tuberculosis* H37Ra (Figure 6), indicating that PZA accumulated to a lesser extent in the PZA-resistant Rv1258c S292L and V219A mutants—which is an indication of higher efflux activity of the Rv1258c mutants—than the parent strain. In addition, the extent of [^14^C]POA accumulation was in accordance with the degree of resistance of the Rv1258c mutants, with the S292L mutant accumulating less POA than the V219A mutant, while both mutants accumulated less POA than the parent strain (Figure 6B).

In conclusion, we demonstrate that point mutations in efflux pump Rv1258c found in clinical isolates can play an important role in conferring clinically relevant drug resistance to multiple drugs including PZA, SM and INH. Our findings could explain some previously unaccounted drug resistance in drug-resistant clinical strains and indicate efflux pump mutations may need to be taken into consideration for improved molecular detection of drug resistance in *M. tuberculosis*. Furthermore, efflux pump inhibitor piperine may be used as adjunct for more effective possible treatment of multi-drug resistant *M. tuberculosis* in future studies.

## Author Contributions

YZ, JL, XH, and VD designed the experiments. JL, WS, SZ, DM, KS, and OB performed the experiments. JL, WS, XH, VD, and YZ analyzed the data. JL and YZ wrote the manuscript.

## Conflict of Interest Statement

The authors declare that the research was conducted in the absence of any commercial or financial relationships that could be construed as a potential conflict of interest.

## References

[B1] Ainsa J. A. Blokpoel M. C. Otal I. Young D. B. De Smet K. A. Martin C. (1998). Molecular cloning and characterization of Tap, a putative multidrug efflux pump present in *Mycobacterium fortuitum* and *Mycobacterium tuberculosis*. *J. Bacteriol.* 180 5836–5843. 9811639 10.1128/jb.180.22.5836-5843.1998 PMC107655

[B2] Andries K. Villellas C. Coeck N. Thys K. Gevers T. Vranckx L. (2014). Acquired resistance of *Mycobacterium tuberculosis* to bedaquiline. *PLoS One* 9:e102135. 10.1371/journal.pone.0102135 25010492 PMC4092087

[B3] Banerjee A. Dubnau E. Quemard A. Balasubramanian V. Um K. S. Wilson T. (1994). inhA, a gene encoding a target for isoniazid and ethionamide in *Mycobacterium tuberculosis*. *Science* 263 227–230. 10.1126/science.8284673 8284673

[B4] Cheng S. J. Thibert L. Sanchez T. Heifets L. Zhang Y. (2000). pncA mutations as a major mechanism of pyrazinamide resistance in *Mycobacterium tuberculosis*: spread of a monoresistant strain in Quebec. *Can. Antimicrob. Agents Chemother.* 44 528–532. 10.1128/AAC.44.3.528-532.2000 10681313 PMC89721

[B5] Cloete R. Kapp E. Joubert J. Christoffels A. Malan S. F. (2018). Molecular modelling and simulation studies of the *Mycobacterium tuberculosis* multidrug efflux pump protein Rv1258c. *PLoS One* 13:e0207605. 10.1371/journal.pone.0207605 30475855 PMC6261026

[B6] Hartkoorn R. C. Uplekar S. Cole S. T. (2014). Cross-resistance between clofazimine and bedaquiline through upregulation of MmpL5 in *Mycobacterium tuberculosis*. *Antimicrob. Agents Chemother.* 58 2979–2981. 10.1128/AAC.00037-14 24590481 PMC3993252

[B7] Heinrichs M. T. May R. J. Heider F. Reimers T. Sy S. K. Peloquin C. A. (2018). *Mycobacterium tuberculosis* strains H37Ra and H37Rv have equivalent minimum inhibitory concentrations to most antituberculosis drugs. *Int. J. Mycobacteriol.* 7 156–161. 10.4103/ijmy.ijmy_33_18 29900893

[B8] Jiang X. Zhang W. Zhang Y. Gao F. Lu C. Zhang X. (2008). Assessment of efflux pump gene expression in a clinical isolate *Mycobacterium tuberculosis* by real-time reverse transcription PCR. *Microb. Drug Resist.* 14 7–11. 10.1089/mdr.2008.0772 18321205

[B9] Kanji A. Hasan R. Ali A. Zaver A. Zhang Y. Imtiaz K. (2017). Single nucleotide polymorphisms in efflux pumps genes in extensively drug resistant *Mycobacterium tuberculosis* isolates from Pakistan. *Tuberculosis* 107 20–30. 10.1016/j.tube.2017.07.012 29050768

[B10] Kardan-Yamchi J. Kazemian H. Haeili M. Harati A. A. Amini S. Feizabadi M. M. (2019). Expression analysis of 10 efflux pump genes in multi/extensively drug-resistant *Mycobacterium tuberculosis* isolates of clinical origin. *J. Glob. Antimicrob. Resist.* 10.1016/j.jgar.2019.01.003 [Epub ahead of print]. 30654147

[B11] Li X. Z. Nikaido H. (2009). Efflux-mediated drug resistance in bacteria: an update. *Drugs* 69 1555–1623. 19678712 10.2165/11317030-000000000-00000 PMC2847397

[B12] Liu J. Takiff H. E. Nikaido H. (1996). Active efflux of fluoroquinolones in *Mycobacterium smegmatis* mediated by LfrA, a multidrug efflux pump. *J. Bacteriol.* 178 3791–3795. 10.1128/jb.178.13.3791-3795.1996 8682782 PMC232638

[B13] Parish T. Stoker N. G. (2000). Use of a flexible cassette method to generate a double unmarked Mycobacterium tuberculosis tlyA plcABC mutant by gene replacement. *Microbiology* 146 1969–1975. 10.1099/00221287-146-8-1969 10931901

[B14] Ramon-Garcia S. Martin C. Thompson C. J. Ainsa J. A. (2009). Role of the *Mycobacterium tuberculosis* P55 efflux pump in intrinsic drug resistance, oxidative stress responses, and growth. *Antimicrob. Agents Chemother.* 53 3675–3682. 10.1128/AAC.00550-09 19564371 PMC2737831

[B15] Ramon-Garcia S. Mick V. Dainese E. Martin C. Thompson C. J. De Rossi E. (2012). Functional and genetic characterization of the tap efflux pump in Mycobacterium bovis BCG. *Antimicrob. Agents Chemother.* 56 2074–2083. 10.1128/AAC.05946-11 22232275 PMC3318366

[B16] Sharma S. Kumar M. Nargotra A. Koul S. Khan I. A. (2010). Piperine as an inhibitor of Rv1258c, a putative multidrug efflux pump of *Mycobacterium tuberculosis*. *J. Antimicrob. Chemother.* 65 1694–1701. 10.1093/jac/dkq186 20525733

[B17] Shi W. Zhang X. Jiang X. Yuan H. Lee J. S. Barry C. E. III (2011). Pyrazinamide inhibits trans-translation in *Mycobacterium tuberculosis*. *Science* 333 1630–1632. 10.1126/science.1208813 21835980 PMC3502614

[B18] Shur K. V. Maslov D. A. Mikheecheva N. E. Akimov N. I. Bekker O. B. Danilenko V. N. (2017). The Intrinsic antibiotic resistance to β-Lactams, macrolides, and fluoroquinolones of mycobacteria Is mediated by the whiB7 and tap Genes. *Russ. J. Genet.* 53 1006–1015. 10.1134/S1022795417080087

[B19] WHO (2018). *Global Tuberculosis Report.* Available at: http://www.who.int/tb/publications/global_report/en/

[B20] Yee M. Gopal P. Dick T. (2017). Missense mutations in the unfoldase ClpC1 of the caseinolytic protease complex are associated with pyrazinamide resistance in *Mycobacterium tuberculosis*. *Antimicrob. Agents Chemother.* 61:e02342-16. 10.1128/AAC.02342-16 27872068 PMC5278685

[B21] Zhang S. Chen J. Shi W. Cui P. Zhang J. Cho S. (2017). Mutation in *clpC1* encoding an ATP-dependent ATPase involved in protein degradation is associated with pyrazinamide resistance in *Mycobacterium tuberculosis*. *Emerg. Microbes Infect.* 6:e8. 10.1038/emi.2017.1 28196969 PMC5322326

[B22] Zhang S. Chen J. Shi W. Liu W. Zhang W. H. Zhang Y. (2013). Mutations in *panD* encoding aspartate decarboxylase are associated with pyrazinamide resistance in *Mycobacterium tuberculosis*. *Emerg. Microbes Infect.* 2:e34. 10.1038/emi.2013.38 26038471 PMC3697303

[B23] Zhang Y. Heym B. Allen B. Young D. Cole S. (1992). The catalase-peroxidase gene and isoniazid resistance of *Mycobacterium tuberculosis*. *Nature* 358 591–593. 10.1038/358591a0 1501713

[B24] Zhang Y. Mitchison D. (2003). The curious characteristics of pyrazinamide: a review. *Int. J. Tuberc. Lung Dis.* 7 6–21. 12701830

[B25] Zhang Y. Scorpio A. Nikaido H. Sun Z. (1999). Role of acid pH and deficient efflux of pyrazinoic acid in unique susceptibility of *Mycobacterium tuberculosis* to pyrazinamide. *J. Bacteriol.* 181 2044–2049. 10094680 10.1128/jb.181.7.2044-2049.1999 PMC93615

[B26] Zhang Y. Shi W. Zhang W. Mitchison D. (2013). Mechanisms of pyrazinamide action and resistance. *Microbiol. Spectr.* 2 1–12. 10.1128/microbiolspec.MGM2-0023-2013 25530919 PMC4268777

[B27] Zhang Y. Yew W. W. (2009). Mechanisms of drug resistance in *Mycobacterium tuberculosis*. *Int. J. Tuberc. Lung Dis.* 13 1320–1330. 19861002

[B28] Zhang Y. Zhang J. Cui P. Zhang Y. Zhang W. (2017). Identification of novel efflux proteins Rv0191, Rv3756c, Rv3008, and Rv1667c involved in pyrazinamide resistance in *Mycobacterium tuberculosis*. *Antimicrob. Agents Chemother.* 61 e940–e917. 10.1128/AAC.00940-17 28584158 PMC5527661

[B29] Zhu Y. H. Gao Y. F. Chen F. Liu W. Zhai M. X. Zhai W. J. (2011). Identification of novel T cell epitopes from efflux pumps of *Mycobacterium tuberculosis*. *Immunol. Lett.* 140 68–73. 10.1016/j.imlet.2011.06.009 21756938

